# Salivary Calculous

**Published:** 1891-03

**Authors:** W. Williams


### Salivary Calculous—Origin and Cause of Accumulation Upon the Teeth.

BY W. WILLIAMS, M.D.

The origin of salivary calculous is undoubtedly the saliva. Saliva is the secretion of two types of glands, the serous or albuminoid and the mucous. The former are situated outside the mouth and include the parotid, submaxillary and sublingual.

The mucous group include the glands and follicles that are situated or imbedded in the walls of the mouth and are the labial buccal, palatine and lingual.

The salivary glands are most active immediately before and during a meal. The mucous glands contribute most of the salvia between the meals.

The reaction of the salivary secretion is alkaline, that of buccal glands slightly acid, and that of mixed saliva slightly alkaline or neutral.

The saliva is composed of about five per cent. of solids, the balance water. The solid portion is made up of the ferment ptyalin, the salts of lime, potassium, sodium and magnesium, with the debris of epithetical scales and food. It is also rich in micro-organisms.

Saliva is a secretion and not an excretion, the latter is of no further use in the economy while the former is. The urine furnishes an example of excretion while saliva is an example of secretion, being of further use as an aid to the digestion of food. The substances found in an excretion are found as such in the blood and it is possible for an excretionary organ to be removed and another organ to take on and perform its unfulfilled function. The secretions and excretions of the body are saturated with the different salts they hold in solution.

These solutions are subject to the same laws, both physical and chemical, that govern solutions outside the body. A saturated solution of common salt or nitrate of potash, for instance, will remain clear until a foreign body is introduced, when crystalization will commence around this nucleus immediately and continue until the whole volume is crystalized.

The saliva is particularly saturated with the salts of lime which have a special tendency to throw down a precipitate. The salts of lime differ from most of the other salts in being more soluble in cold than in hot water. The formation of calculi is rather common in all the secretions and excretions, particularly in the gall-bladder, where they are known as biliary calculi. In the kidneys and bladder they also occur and occasionally a calculous is found in the salivary glands or their ducts. Biliary calculi is found most generally in females and men of sedentary habits, perhaps accounted for from the precipitation of some of the elements of the bile in the gall-bladder from mere sluggishness. It is thus seen that the most guarded and best protected secretions are liable to have this calculous formation. Is it then any wonder that a deposition of calculous upon the teeth is common when we remember the composition of the saliva and the changes it is exposed to in the mouth. Every experiment is here played with it, freezing and boiling liquids, acids, alcohol, and a long test of other substances too numerous to even mention them, are daily mixed with it.

Disease, constitutional and local, want of use, physical and chemical causes, any one of these might vitiate, disturb and change the nature of normal saliva and cause a precipitation. We must not forget the micro-organisms that play such an important part in the saliva, which soon, when at rest, is rendered acid by the fermentation products due to their activity, the chief source of acid present in the mouth being due to these organisms. This fermentation takes place rapidly wherever the conformation of the teeth allows the process to be undisturbed.

Thus we see that there are various conditions always present that might cause a precipitation in the saliva. The teeth form such splendid nucleus for this precipitation when once formed to be deposited upon, where unfortunately it is often allowed to remain undisturbed.

The composition of salivary calculous is roughly :

| | |
|---|---|
| Lime Salts | 80 parts. |
| Mucous | 12 " |
| Ptyalin | 1 " |
| Organic Matter | 7 " |

The composition of the calculous varies in different parts of the mouth, as for instance, it is stated that that opposite the parotid contains most carbonate, while that opposite submaxillary most phosphate of lime.

